# Linking physiological approaches to marine vertebrate conservation: using sex steroid hormone determinations in demographic assessments

**DOI:** 10.1093/conphys/cot035

**Published:** 2014-02-11

**Authors:** Vanessa Labrada-Martagón, Tania Zenteno-Savín, Marc Mangel

**Affiliations:** 1 Center for Stock Assessment Research, University of California, Santa Cruz, Santa Cruz, CA 95064, USA; 2 Programa de Planeación Ambiental y Conservación, Centro de Investigaciones Biológicas del Noroeste, S.C., La Paz, Baja California Sur, México C.P. 23096; 3 Department of Biology, University of Bergen, Bergen 5020, Norway

**Keywords:** Conservation, life history, marine vertebrates, sex steroids, stock assessment

## Abstract

Sex, age and sexual maturation are key biological parameters for aspects of life history and are fundamental information for assessing demographic changes and the reproductive viability and performance of natural populations under exploitation pressures or in response to environmental influences. Much of the information available on the reproductive condition, length at sexual maturity and sex determinations of endangered species has been derived from direct examination of the gonads in dead animals, either intentionally or incidentally caught, or from stranded individuals. However, morphological data, when used alone, do not provide accurate demographic information in sexually monomorphic marine vertebrate species (e.g. sharks, sea turtles, seabirds and cetaceans). Hormone determination is an accurate and non-destructive method that provides indirect information about sex, reproductive condition and sexual maturity of free-ranging individuals. Correlations between sex steroid concentrations and biochemical parameters, gonadal development and state, reproductive behaviour and secondary external features have been already demonstrated in many species. Different non-lethal approaches (e.g. surgical and mark–recapture procedures), with intrinsic advantages and disadvantages when applied on free-ranging organisms, have been proposed to asses sex, growth and reproductive condition. Hormone determination from blood samples will generate valuable additional demographic information needed for stock assessment and biological conservation.

## Abstract (precis)

Hormone determination is an accurate and non-destructive method that provides demographic information concerning populations of long lived in which sex and maturity are impossible to assess by non-lethal, visual/external examination. We discuss the questions that have already been addressed with these methods, the advantages and disadvantages of them, and the demographic knowledge that can be derived for marine megafauna using hormone determination.

## Introduction

Biological parameters describing key aspects of life history, such as sex, age structure, the dynamics of growth and sexual maturation (recruitment for breeding), are generally considered fundamental information for assessing demographic changes, reproductive viability and the performance of natural populations under exploitation pressures or in response to environmental influences, for establishing the accurate conservation status of free-ranging populations and for developing conservation and sustainable fisheries management strategies (DeMaster, 1984; Perrin and Reilly, 1984; Eberhardt, 1985; Balazs, 1995; Crouse and Frazer, 1995; Haddon, 2001; Heppell *et al.*, 2003; de Mitcheson, 2009; alternative approaches are described by Kirkwood, 1991; Cook, 1995; Punt and Donovan, 2007). However, certain basic information needed to predict the population dynamics of threatened species is particularly difficult to obtain solely by visual determination. This is especially true for sexually monomorphic marine vertebrate wildlife (e.g. sharks, seabirds, cetaceans and sea turtles; Caldwell, 1962; Bercovitz *et al.*, 1978; Owens *et al.*, 1978; Merchant-Larios, 1999; Wibbels *et al.*, 2000; Bertellotti *et al.*, 2002; Casale *et al.*, 2005; Dubiec and Zagalaska-Neubauer, 2006). Consequently, much of this information has been derived from dead animals, either directly or incidentally caught, or from stranded individuals (Hohn *et al.*, 1985; Read, 1990; Valencia and Leyton, 1992; Merchant-Larios, 1999; Kellar *et al.*, 2009; Casale *et al.*, 2011). Even when data can be obtained directly from free-ranging animals by photo-identification or tagging studies, the population estimates for wild marine fauna are complicated by the difficulties of capturing and tagging, the effects of tagging, the recovery rate and the requirement for long-term monitoring series (Gibbons, 1987; Clapham, 1992; Limpus, 1992; Balazs, 1995, 1999; Balazs and Chaloupka, 2006; Dugger *et al.*, 2006).

Measurement of hormone concentrations provides an alternative methodology that can be used for understanding physiological cycles, functional aspects of reproductive endocrinology and the relationship between the hormone dynamics and the reproductive and nesting behaviour (e.g. Licht *et al.*, 1979, 1980, 1985; Owens, 1980; Licht, 1982; Owens and Morris, 1985; Wibbels *et al.*, 1990, 1992; Williams, 1992a; Al-Habsi *et al.*, 2006; Awruch *et al.*, 2008b). Recent publications have demonstrated the utility of hormone measurement as a non-destructive method to obtain information on the reproductive condition of endangered species without conspicuous sexual dimorphism (Rolland *et al.*, 2005; Daoquan *et al.*, 2006; Awruch *et al.*, 2008a; Kellar *et al.*, 2009; Valente *et al.*, 2011). Currently, hormone biomarkers are sufficiently accurate to provide information about overall health (e.g. biochemical parameters and moult) in response to environmental (e.g. season and El Niño event) and regional conditions (e.g. tourism and chemical contaminants; Romero and Wikelski, 2001, 2002a,b; Mashburn and Atkinson, 2004; Oki and Atkinson, 2004; Labrada-Martagón *et al.*, 2013). Such biomarkers thus contribute valuable information on the physiological responses of wildlife to xenobiotics and environmental change (Guillette *et al.*, 1994, 1999; Guillette and Gunderson, 2001; Hall *et al.*, 2003).

Vertebrates have evolved neuroendocrine signals, patterns of sexual development and complex behavioural responses (social and sexual behaviours mediated by the nervous system) that are, in general, highly conserved across taxa, including elasmobranchs (Callard *et al.*, 1989; Crews *et al.*, 1994; Romero, 2004; Pratt and Carrier, 2005); two examples are the stress response (to potentially dangerous stimuli) and the sex steroid and thyroid hormone control systems (Randall *et al.*, 1997; Wingfield *et al.*, 1997; Romero, 2002; Willmer *et al.*, 2005; Rastogi, 2007). There are, of course, exceptions found in amphibians and reptiles (Dickhoff and Darling, 1983; Willmer *et al.*, 2005), such as temperature-dependent sex determination (Crews *et al.*, 1994). Due to the need to match research with conservation measures and the need for accurate data and understanding, a main current goal for ecologists is the development and application of less invasive tools for physiological studies of threatened wildlife (Bercovitz *et al.*, 1978; Hunt *et al.*, 2004; Mashburn and Atkinson, 2004; Millspaugh and Washburn, 2004; Sanvito *et al.*, 2004; Bush *et al.*, 2005; Palme, 2005). The interpretation of the health and physiological condition of vertebrates could be seriously affected by the acute physiological stress generated by the capture, restraint and handling conditions. For example, variation in haematocrit, haemoglobin concentration, serum sex steroid concentrations and other biochemical variables (e.g. aspartate aminotransferase activity and levels of glucose, triglycerides and lactate) have been correlated with the handling/sampling time (the time elapsed between capture of the animal and blood sampling) and with cortisol and corticosterone concentrations (Jessop *et al.*, 1999, 2002, 2004b,c; Franklin *et al.*, 2003; Labrada-Martagón *et al.*, 2010) derived from the hypothalamic–pituitary–adrenal axis response (Romero, 2004).

In this review, we reinforce the contribution and relevance of physiological studies as a complement to the more standard demographic data of many threatened and endangered populations of marine vertebrate species that are needed for accurate management and conservational strategies. We give particular attention to those long-lived, monomorphic marine species in which sex and maturity are impossible to assess by non-lethal, visual/external examinations. We discuss the questions that have already been addressed and the demographic knowledge derived from analytical determination of hormone concentrations in marine megafauna. We briefly review the more invasive techniques and procedures that have generated demographic data and population parameters in marine vertebrates, specifically those focused on establishing sex and reproductive status. We then summarize the information derived from each method, as well as the advantages and disadvantages of these techniques, with concluding remarks focusing on the need for wide implementation of hormone determination as a valuable, precise and non-lethal tool for those marine species for which demographic data are lacking.

## The journey from lethal take to non-invasive ecological data

### Steroid hormone determinations

At present, the value (informational, educational, economical and ecological) that a live free-living animal has in the wild is generally accepted (Stonehouse, 1990; Ávila and Saad, 1998; Carwardine and Watterson, 2002; Bjorndal and Jackson, 2003); however, it has not always been so. As researchers began to understand the negative effects that some techniques (e.g. branding, mutilation, banding and surgery) could have on the wellbeing, health, survival and fitness of wildlife under study (Hammond, 1990; Dugger *et al.*, 2006; Mellish *et al.*, 2007; Petrauskas *et al.*, 2008), they moved towards evaluation and application of less invasive methods (Hammond, 1990; Fossi *et al.*, 1992; Jensen *et al.*, 2003; Hunt *et al.*, 2004; Mashburn and Atkinson, 2004; Millspaugh and Washburn, 2004; Sanvito *et al.*, 2004; Bush *et al.*, 2005; Al-Habsi *et al.*, 2006; Sulikowski *et al.*, 2006, 2012; Awruch *et al.*, 2008a; Kellar *et al.*, 2009). One of the breakthroughs occurred in the 1970s, when sea turtle researchers confirmed that serum hormone determination by radioimmunoassay provided equivalent information on the reproductive endocrinology of these organisms without the need to kill the animals, as had been done until then (Owens *et al.*, 1978; Licht *et al.*, 1979, 1980, 1985; Licht, 1980, 1982).

Considerable effort in the study of reproductive endocrinology (the relationship between hormone concentrations and reproductive behaviour) has been focused on adult seabirds (Groscolas *et al.*, 1986; Hector *et al.*, 1986a,b; Williams, 1992a; Williams and Sharp, 1993; Cherel *et al.*, 1994; Mauget *et al.*, 1994; Lormée *et al.*, 2000), mature sharks (Manire *et al.*, 1995; Manire and Rasmussen, 1997; Heupel *et al.*, 1999) and nesting sea turtles (see reviews by Owens, 1997; Hamann *et al.*, 2003).

There is still a lack of understanding about some aspects of endocrine dynamics and their relationship with reproductive ecology, which limits the conclusions that can be drawn about reproductive status when steroid hormone concentrations are used alone. An example is provided by deferred-breeding seabirds, in which physiologically mature individuals with functional gonads and adult hormone levels do not manifest observable breeding events (Williams, 1992b). Variations in the concentrations of gonadotrophins have been evaluated together with steroid hormones in an attempt to understand deferred breeding in penguins (Williams, 1992a, 1992b). Other physiological biomarkers, such as vitellogenin and lipoproteins, which are precursors of egg yolk, could be explored in the future to evaluate reproductive status in seabirds, as has been done in sea turtles (Herbst *et al.*, 2003; Valverde *et al.*, 2008) and birds (Vanderkist *et al.*, 2000). Estrone has also been evaluated and is suggested to be the primary natural estrogen circulating in plasma of adult female sea turtles (Coufal *et al.* 2003; Coufal and Whittier, 2003), given the minimal or undetectable estradiol-17β (E) concentrations reported throughout the nesting period in some female green sea turtle populations (Licht *et al.*, 1980; Wibbels *et al.*, 1992; Al-Habsi *et al.*, 2006).

Hormone measurements can provide indirect clues about the reproductive ecology or behavioural status of marine organisms without the necessity for killing the organisms under study and without the support of additional information that is difficult to obtain by other direct means (e.g. gonad biopsies; Bercovitz *et al.*, 1978; Owens *et al.*, 1978; Wibbels *et al.*, 1987a,b; Gross *et al.*, 1995; Schwarzenberger *et al.*, 1996; Owens, 1997, 1999; Mansour *et al.*, 2002; Rolland *et al.*, 2005; Awruch *et al.*, 2008a; Kellar *et al.*, 2009; Xia *et al.*, 2011). Thus, despite some limitations, steroid hormones [particularly testosterone (T), progesterone (P) and E], measured in non-lethal samples (e.g. blood, amniotic fluid, eggs, blubber, faeces and saliva), have been proposed as an alternative tool for the assessment of sex identification, the reproductive status (gonadal activity and pregnancy), stage of maturity and size at maturity of wild threatened and endangered species. However, until the analytical process is well established and the natural variations and functional differences (e.g. season and sex) of hormone concentrations are well understood for the population of interest, some validating technique (histology, laparoscopy, ultrasound or photo-identification) has to be applied to a small subset of the sampled organisms of known sex or reproductive condition (Wibbels *et al.*, 1987a, 2000; Gross *et al.*, 1995; Wibbels, 1999, 2003; Rolland *et al.*, 2005; Braun-McNeill *et al.*, 2007; Blanvillain *et al.*, 2008; Kellar *et al.*, 2009; Sulikowski *et al.*, 2012). This guarantees accurate interpretation of the information derived from the first and subsequent hormone determinations.

#### Sharks: puberty and sexual maturity

Sharks are the model species in which the hormonal changes (serum T, E and P) have been evaluated during growth (differences between pre-maturity stages) and related to the process of sexual maturity (elevated E and T levels), providing information about the sex steroid changes related to puberty and sexual maturity. These findings complement the well-known relationship of the sex steroids with other reproductive conditions, such as the pre-ovulatory stage, mating process and spawning cycle (Koob *et al.*, 1986; Rasmussen and Gruber, 1993; Manire *et al.*, 1995; Gelsleichter *et al.*, 2002; Sulikowski *et al.*, 2005, 2006; Awruch *et al.*, 2008a,b). Even so, the information about reproductive endocrinology in sharks remains incomplete (Rasmussen and Gruber, 1993; Gelsleichter *et al.*, 2002).

The studies on skates and sharks [e.g. white shark (*Carcharodon carcharias*), draughtboard shark (*Cephaloscyllium laticeps*) and winter (*Leucoraja ocellata*) and thorny (*Amblyraja radiate*) skates] have emphasized that understanding the endocrine dynamics (plasma E, T and P levels) involved during puberty and maturation would provide the opportunity to create an accurate, validated, non-lethal tool for reproductive information, such as the determination of size at sexual maturity, in free-living organisms (Sulikowski *et al.*, 2005, 2006, 2012; Awruch *et al.*, 2008a,b). During growth of the individual, sharks and skates show increased levels of serum T and E that have been associated with some anatomical changes of the gonads, such as annual testicular development, increased size/weight of the testes, ovarian mass, follicle size and shell gland mass (Callard *et al.*, 1989; Manire and Rasmussen, 1997; Sulikowski *et al.*, 2005, 2006). In bonnethead sharks (*Sphyrna tiburo*), differences in serum androgen levels are evident (*P* < 0.05) between stages of maturity in males (early pubertal vs. full gonadal maturity); nevertheless, quantitative correlations between the increase in hormone concentrations and gonadal maturity observed (testis size) were not evaluated (Gelsleichter *et al.*, 2002). Likewise, T concentrations measured in plasma of draughtboard shark males, as well the gonadosomatic index, have shown a significant increase from the juvenile to the adult stage; however, the correlation between the androgen levels and the histological index was not reported (Awruch *et al.*, 2008b). In winter and thorny skates, in which morphological, histological and hormone concentration data were combined to establish age and size at sexual maturity, the concentration of T in plasma of males is directly correlated to clasper length (*r* > 0.79), the percentage of mature spermatocysts (*r* > 0.74) and testis mass (*r* > 0.79) throughout the process of sexual maturity, while female skates show a significant correlation between the E concentration and ovarian mass (*r* > 0.82), shell gland mass (*r* > 0.80) and average follicle size (*r* > 0.80; Sulikowski *et al.*, 2005, 2006).

Awruch *et al.* (2008a) demonstrated that plasma sex steroid determination is a non-lethal technique that provides accurate information about the stage of maturity (juveniles or adults) and size at maturity of chondrichthyans. They reported minor differences (<2%) between estimates of size at maturity based on lethal (gonadal condition) vs. non-lethal approaches (models based on hormone concentrations). The concentrations of T and E, together with total length, allowed them to discriminate the state of maturity of female draughtboard sharks, while T and clasper length together enable discrimination between juvenile and adult male sharks. The T and E provided 90% accuracy when used to classify the reproductive state of female sharks (juveniles and adults), and the T combined with clasper length showed an accuracy of 97% for adult males when validated by gonad examination (Awruch *et al.*, 2008a).

#### Sea turtles: sex identification and reproductive status

For sea turtles, the advantage of using hormone determination, measured in non-lethal biological samples, has been sex identification during stages without evident morphological differences (Owens *et al.*, 1978; Owens, 1997). The concentration of circulating T (immature individuals) and T measured in amniotic fluid (hatchlings) has been broadly used to determine sex in loggerhead sea turtles (*Caretta caretta*), green sea turtles (*Chelonia mydas*), Kemp's ridley sea turtles (*Lepidochelys kempii*) and hawksbill sea turtles (*Eretmochelys imbricata*), due to the lack of morphological differences between sexes at these age stages. The T and E concentrations showed significant differences between sexes in these species, facilitating the use of the E:T ratio for sex determination of the immature sea turtles (Owens *et al.*, 1978; Wibbels *et al.*, 1987a,b, 2000; Gross *et al.*, 1995; Gregory and Schmid, 2001; Braun-McNeill *et al.*, 2007; Xia *et al.*, 2011). The E:T ratio, measured in either plasma or amniotic fluid, provided an overall accuracy of 96% when validated by histological examinations, resulting in a more accurate sexing method than T or E concentrations when used alone (71–86.7% overall accuracy) in *Caretta* and *Chelonia* (Gross *et al.*, 1995; Xia *et al.*, 2011). Immature green sea turtles, however, do not always show clear differentiation patterns in T concentrations between sexes. Exceptions appear to be 4-year-old individuals from a Cayman turtle farm (Owens *et al.*, 1978), artificially incubated hatchlings from China (Xia *et al.*, 2011) and free-ranging Hawaiian immature turtles (not validated by a direct approach; Wibbels *et al.*, 1993). The overlap in ranges of circulating T between sexes means that the use of T for sex identification and sex ratio in green sea turtle juveniles is problematic; other complementary visual methods have been recommended (Bolten *et al.*, 1992; Wibbels *et al.*, 2000; Labrada-Martagón, 2011).

In addition to physiological cycles, reproductive endocrinology and the relationship between the hormone dynamics and reproductive and nesting behaviours in female adults (e.g. Licht *et al.*, 1979, 1980, 1985; Owens, 1980; Licht, 1982; Owens and Morris, 1985; Wibbels *et al.*, 1990, 1992; Williams, 1992a; Al-Habsi *et al.*, 2006), the evaluation of T concentration in plasma has been used to determine the reproductive status (active breeders vs. inactive ones) of adult male loggerhead sea turtles in order to gain information about their reproductive biology. Differences in plasma T concentrations have been reported between males with different reproductive behaviour and testis condition, with males classified as active breeders showing higher levels than non-breeders (Jessop *et al.*, 2004a; Blanvillain *et al.*, 2008). The T concentration measured in plasma provided 100% accuracy in discriminating the reproductive status of adult male loggerheads when validated with laparoscopy; here, a level of T <5 ng ml^−1^ is considered to indicate a reproductively inactive male (Blanvillain *et al.*, 2008). Increased levels of circulating T have been observed before the mating season in loggerhead and green sea turtle males, suggesting testicular development and a peak in spermatogenesis (Licht *et al.*, 1985; Wibbels *et al.*, 1990; Blanvillain *et al.*, 2008). The concentration of T in plasma has been directly correlated with an increase in the seminiferous tubule diameter in the loggerhead turtles (Pearson's *r* = 0.65, *P* < 0.0005; Wibbels *et al.*, 1990; Blanvillain *et al.*, 2008). Seasonal changes in circulating levels of T in sea turtles, related to the testicular recrudescence and spermatogenesis in adult males (Licht *et al.*, 1985; Wibbels *et al.*, 1987a, 1990; Jessop *et al.*, 2004a), could be a confounding factor during the determination of sex ratios of adults. The accuracy of T levels in plasma, when used as a sexing method in immature loggerheads, decreased by 67% from summer to winter due to the overlapping values between females and males observed during the non-breeding period (Braun-McNeill *et al.*, 2007). For that reason, for sexual determination it is recommended to use those blood samples taken during the summer, when the elevated values of T are found in loggerheads (Braun-McNeill *et al.*, 2007; Blanvillain *et al.*, 2008).

#### Sea turtles and seabirds: resource allocation and deferred sexual maturity

The evaluation of circulating hormone concentrations has helped in the identification of the environmental (e.g. photoperiod) and energetic cues (e.g. body condition and lipid reserves) that stimulate gonadal development, migration, nesting behaviour and reproduction in seabirds (both sexes) and sea turtles (females). The E concentration is closely related to the maturation of the ovarian follicles and vitellogenesis (Groscolas *et al.*, 1986; Hector *et al.*, 1986b; Hamann *et al.*, 2003). The elevated levels of plasma steroids (E and T) and triglycerides and the increase in follicular and testicular size observed before migration, courtship and the pre-laying period in sea turtles and seabirds all suggest that resource allocation (metabolic preparation), follicular development and elevated activity of the gonads occur before arrival at the breeding grounds (Groscolas *et al.*, 1986; Hector *et al.*, 1986b; Hamann *et al.*, 2002a, 2002b, 2003).

In Adélie (*Pygoscelis adeliae*) and Emperor penguins (*Aptenodytes forsteri*), the increase in plasma T concentration observed in males at the time of copulation has been related to the period of testicular development and spermatogenesis (Groscolas *et al.*, 1986). In male and female black-browed (*Diomedea melanophris*), grey-headed (*D. chrysostoma*) and wandering albatross (*D. exulans*), the highest levels of sex steroids in plasma can be found during the pre-laying period. In these species of seabirds, sexual differences in the dynamics of circulating P concentration can be found during the breeding cycle (Hector *et al.*, 1986a,b).

Williams (1992b) explained deferred sexual maturity in Macaroni (*Eudyptes chrysolophus*) and Gentoo penguins (*Pygoscelis papua*) by variation of the plasma steroids, and concluded that delays in breeding should be related to behavioural or ecological factors instead of an immature physiological state, because late breeder male and female penguins were physiologically mature at 5 years and 1 year, respectively, prior to their first breeding event. Immature wandering albatross, which also exhibit deferred sexual maturity, have shown a significant increase in testicular size and plasma T concentration with age; even when no differences in the size of the gonads were found, immature individuals that had not yet bred, older than 5 years, showed lower concentrations of T than breeders. Albatross 10 years old were the group of immature birds (not breeders) that did not show differences in the concentration of T with respect to adults (Hector *et al.*, 1986a).

#### Cetaceans: maturity and reproductive condition

In marine mammals, the study of sex steroids has mainly involved cetaceans [e.g. right whales (*Eubalaena glacialis*), common dolphins (*Delphinus delphis*) and finless porpoise (*Neophocaena phocaenoides*)] to assess reproductive stage (juveniles), status (pregnancy and lactation), seasonality, sex and maturity (Mansour *et al.*, 2002; Rolland *et al.*, 2005; Daoquan *et al.*, 2006; Greig *et al.*, 2007; Hao *et al.*, 2007; Kellar *et al.*, 2009). The concentration of T in serum has been used to describe the maturity condition (immature, pubescent and fully mature) and age at maturity of a captive finless porpoise male, in which the pattern of growth in body length coincided with the pattern of increase of the concentration of serum T measured in the individual (Daoquan *et al.*, 2006). Blubber concentrations of T have been compared between stages of maturity in common dolphins; the highest T concentrations occur in mature dolphins, compared with pubertal and immature individuals. The concentration of T measured in adipose tissue biopsies taken from free-swimming common dolphins have not shown significant differences when compared with values measured in stranded and incidentally captured individuals of known stages of maturity. This suggests the utility of blubber T concentration to assess maturity condition in this species (Kellar *et al.*, 2009). In the North Atlantic right whale (*E. glacialis*), the faecal ratio of androgen to estrogen concentration is an accurate approach for sex determination; this has been validated with photo-identified individuals (Rolland *et al.*, 2005). Faecal hormone concentrations have been suggested as an additional tool for assessing sexual maturity and reproductive state in free-ranging right whales. The concentration of faecal T showed significant differences between males when grouped according to maturity status (immature vs. adult). Pregnant and lactating right whale females were also identifiable from other female maturity subgroups (juvenile or resting adult) according to specific faecal hormone profile characteristics (concentrations of progestins, estrogens and androgens; Rolland *et al.*, 2005). The lack of correlation between the weight of the faecal sample taken and the hormone concentrations measured (Rolland *et al.*, 2005) guarantees the accuracy when using this approach in cetaceans. We lack additional information on sex hormone concentrations in marine mammals because of the difficulties inherent in obtaining blood or faecal samples in free-living cetaceans (Daoquan *et al.*, 2006; Rolland *et al.*, 2006; Kellar *et al.*, 2009).

#### Additional advantages of steroid hormone determinations

Circulating hormone determination in endangered wildlife has additional benefits. In the field, the capture and handling protocol is limited to blood sampling (Wibbels, 1999), which can be completed in <15 min (Jessop *et al.*, 1999, 2002, 2004c; Blanvillain *et al.*, 2008), thus facilitating the collection of a large number of samples with minimal stress to the individuals. Sex steroids are fairly stable metabolites, so serum samples can be stored frozen (−20°C) for long periods (Wibbels, 1999) without using a large amount of laboratory space. The analytical procedures used to determine the hormone concentrations (radioimmunoassay and enzyme-linked immunosorbent assay) are relatively inexpensive, large numbers of samples can be analysed simultaneously in a short period of time, and the analytical protocols have been extensively described for many marine species. Thus, hormone determination is a practical and inexpensive means for evaluating the demographic data of large numbers of individuals. The type of sample should be chosen according to the species of interest, thus facilitating the field work (e.g. blubber or faecal samples in cetaceans; amniotic fluid in hatchlings). This technique could be assessed for younger and immature individuals, such as calves, chicks and hatchlings; in the case of immature sea turtles, it could be used in individuals as short as 25 cm of straight carapace length (Wibbels, 1999).

### Comparison with classical approaches for assessment of population parameters

The most common procedures used to determine sex, sexual maturation and reproductive condition of marine megafauna have been the following: (i) direct examination of gonads and hard body parts; (ii) mark–recapture monitoring and photo-identification; and (iii) visual examination of external morphology (Table 1). The analysis of growth marks in hard body parts generates estimates of age, growth rates and some insights about the age–length and age at maturity relationships (Table 1). Such data may also be helpful in understanding the relationship between skeletal development (growth) and physiological maturity in wildlife (e.g. Campana *et al.*, 2002; Natanson *et al.*, 2002; Zug *et al.*, 2002; Luque *et al.*, 2007; Avens *et al.*, 2009; Goshe *et al.*, 2010). The main limitation is that these techniques are only accurate when recovered carcasses are validated with known-age data (e.g. Bowen *et al.*, 1983; Kusher *et al.*, 1992; Bjorndal *et al.*, 1998; Natanson *et al.*, 2002; Snover *et al.*, 2011).

**Table 1.** Classical, direct approaches for population parameter assessment employed for marine megafauna

Method	Megafauna group	Species examples	Demographic data	Disadvantages	References
Histological examination of gonads	Pelagic sharks Penguins Sea turtles Dolphins	Blue shark (*Prionace glauca*) Whitetip shark (*Carcharhinus longimanus*) *Pygoscelids* penguins Green sea turtle (*Chelonia mydas*) Loggerhead sea turtle (*Caretta caretta*) Olive Ridley sea turtle (*Lepidochelys olivacea*) Leatherback sea turtle (*Dermochelys coriacea*) Spotted dolphin (*Stenella attenuata*) Bottlenose dolphin (*Tursiops truncatus*) Harbour porpoise (*Phocoena phocoena*)	Reproductive biology/anatomy Reproductive condition Gonadosomatic index Sex determination Sex ratio Sexual maturity Age/size at maturity Gonadal condition Maturity status Age classes Breeding period Seasonality Number of embryos Fecundity/pregnancy rate Size range of gravid individuals Age–length relationship	Lethal approach Requires carcass recovery Incidental captures by fisheries nets Stranded individuals Requires adequately equipped laboratory Requires experience for detailed morphological observation and accurate interpretation Specific variations in the degree of gonadal differentiation at hatching (sea turtles)	Strasburg (1958); Owens (1980); Stevens (1983); Perrin and Reilly (1984); Hohn *et al*. (1985); Natanson and Cailliet (1986); Callard *et al*. (1989); Peddemors (1989); Read (1990); Kusher *et al*. (1992); Valencia and Leyton (1992); Gross *et al*. (1995); Merchant (1999); Hazin *et al*. (2001); Blanvillain *et al*. (2008); de Mitcheson (2009); Xia *et al*. (2011)
Analysis of growth marks in hard body parts: Dentine deposition rate Ossification patterns Vertebral annuli (band) counts Skeletochronology (humerus and scleral ossicle bones)	Pelagic sharks Earless seals Sea turtles Cetaceans	Leopard shark (*Triakis semifasciata*) Porbeagle shark (*Lamna nasus*) Shortfin mako (*Isurus oxyrinchus*) Blacktip shark (*Carcharhinus limbatus*) Spinner shark (*Carcharhinus brevipinna*) Green sea turtle (*Ch. mydas*) Loggerhead sea turtle (*C. caretta*) Leatherback sea turtle (*D. coriacea*) Harp seal (*Phoca greonlandica*) Beluga (*Delphinapterus leucas*) Dall's porpoise (*Phocoenoides dalli*) Short-finned pilot whale (*Globicephala macrorhynchus*) Bottlenose dolphin (*T. truncatus*)	Age Growth rates Age–length relationship Age at maturation Skeletal development (growth) and physiological maturity relationship	Requires carcass recovery or bone biopsies Need for validation with known-age data Assumptions about: Deposition rates (annual) Recognition of layers Interpretation of layers Loss of layers Remodelling of bone and resorption of layers with growth (sea turtles) Requires correction factors in sea turtles (ectotherms)	Ogden *et al*. (1981); Bowen *et al*. (1983); Zug *et al*. (1986, 2002); Branstetter (1987); Peddemors (1989); Read (1990); Zug (1990); Kusher *et al*. (1992); Parham and Zug (1997); Bjorndal *et al*. (1998); Zug and Glor (1998); Campana *et al*. (2002); Natanson *et al*. (2002); Luque *et al*. (2007); Avens *et al*. (2009); Goshe *et al*. (2010); Snover *et al*. (2011)
Laparoscopy	Sea turtles	Green sea turtle (*Ch. mydas*) Loggerhead sea turtle (*C. caretta*)	Sex determination Reproductive condition Maturity status Diagnosis of pathological processes and anatomical anomalies (avian species)	Surgical technique Invasive and stressful Impractical for sex determination in penguins Not applicable in early stages of development Requires general (birds) or local anaesthetic (sea turtles) Could compromise the individual's health: Trauma to vital organs Potential infections Requires previous clinical and physical examination of the health state of the individuals Logistically difficult and time consuming in the field Requires specialized equipment and scientists/veterinarians with proper training Expensive	Samour *et al*. (1983); Wood *et al*. (1983); Wibbels *et al*. (1987b, 1990, 2000); McDonald (1996); Owens (1999); Wibbels (1999); Jessop *et al*. (2004a); Braun-McNeill *et al*. (2007); Cerit and Avanus (2007); Blanvillain *et al*. (2008); Costantini *et al*. (2008); Matta *et al*. (2008)
Cloacascopy	Penguins	Adélie penguin (*Pygoscelis adeliae*) Chinstrap penguin (*Pygoscelis antarctica*) Humboldt penguin (*Spheniscus humboldti*)	Sex determination	Requires structures well differentiated in size Not applicable to chicks Applicable only in individuals older than 11 months Accuracy is compromised towards the end of the breeding season Specific variations in cloacae characteristics are difficult to assess in the field Applicability is dependent on field conditions Requires specialized equipment and trained experts	Ainley *et al*. (1983); Samour *et al*. (1983); Boersma and Davies (1987); Kerry *et al*. (1992); Zavalaga and Paredes (1997); Costantini *et al*. (2008)
Ultrasonography	Sea turtles Cetaceans	Kemp's Ridley sea turtle (*Lepidochelys kempi*) Loggerhead sea turtle (*C. caretta*) Bottlenose dolphin (*T. truncatus*)	Reproductive condition Sex determination	External, non-invasive, promising tool Requires further validation studies Requires complete gonadal maturation (follicle size) Not useful for sex determination in immature individuals (sea turtles) Requires specialized equipment Requires anatomical knowledge for accurate interpretation Time consuming per individual examination	Rostal *et al*. (1990, 1997); Brook *et al*. (2000); Brook (2001); Valente *et al*. (2007); Blanvillain *et al*. (2008)
Mark–recapture monitoring: Tagging Photo-identification of natural marks	Penguins Sea turtles Cetaceans	Adélie penguin (*P. adeliae*) Emperor penguin (*Aptenodytes forsteri*) Magellanic penguin (*Spheniscus magellanicus*) Blue penguin (*Eudyptula minor*) Yellow-eyed penguin (*Megadyptes antipodes*) Humboldt penguin (*S. humboldti*) African penguin (*Spheniscus demersus*) Rockhopper penguin (*Eudyptes chrysocome*) Green sea turtle (*Ch. mydas*) Loggerhead sea turtle (*C. caretta*) Hawksbill turtle (*Eretmochelys imbricata*) Bottlenose dolphin (*T. truncatus*) Spotted dolphin (*Stenella frontalis*) Pilot whale (*Globicephala melaena*) Humpback dolphin (*Sousa chinensis*) Right whale (*Eubalaena australis*) Humpback whale (*Megaptera novaeangliae*) Killer whale (*Orsinus orca*) Blue whale (*Balaenoptera musculus*) Fin whale (*Balaenoptera physalus*) Bryde's whale (*Balaenoptera edeni*) Minke whale (*Balaenoptera acutorostrata*) Bowhead whale (*Balaenoptera mysticetus*) Sperm whale (*Physeter macrocephalus*) Gray whale (*Eschrichtius robustus*) Manatee (*Trichechus manatus*)	Reproductive/breeding cycles Reproductive condition Sex determination Growth rates Age at sexual maturity Age class determination Age at fist pregnancy/birth Fecundity rate Reproductive success Survival and reproductive probability estimates Copulation, incubation and attendance pattern behaviours Migratory routes and periodicity between habitats Philopatry and site fidelity Population size/rate change Population identity/subunit and family group identification Feeding and breeding habitats	Long-term monitoring series (years) High economic cost Tagging: Absence of suitable tags for specific age classes Tagging is dependent on field conditions Accessibility to habitats (feeding grounds) Tagging confined to specific: Age classes Sex Life stages/size distribution Low rate of recaptures Tag loss Photo-identification: Changes in marks over time Similar patterns between individuals/twins Lack of clearly defined notch Process of recognition and matching is time consuming Probability of identification error Expensive photographic equipment	Ainley and DeMaster (1980); Ainley *et al*. (1983); Bjorndal *et al*. (1983); Carr (1984); Groscolas *et al*. (1986); Boersma *et al*. (1990); Dann and Cullen (1990); Darby and Seddon (1990); Hammond (1990); Clapham (1992); Limpus (1992); Green (1993); Balazs (1995, 1999); Crouse and Frazer (1995); Meylan (1995); Chaloupka and Musick (1997); Herzing (1997); Zavalaga and Paredes (1997); Karczmarski and Cockcroft (1998); Crawford *et al*. (1999); Bjorndal *et al*. (2000); Seminoff *et al*. (2002); Grellier *et al*. (2003); Balazs and Chaloupka (2004); Chaloupka *et al*. (2004); Kendall *et al*. (2004); Langtimm *et al*. (2004); Dugger *et al*. (2006); Richardson *et al*. (2006); Koch *et al*. (2007); Braun-McNeill *et al*. (2007); Lopez-Castro *et al*. (2010); Poisbleau *et al*. (2010)
Discriminant functions based on biometric data (seabirds): Bill, foot, wing and vent measurements Visual examinations of phenotypical characteristics: Clasper morphology Mean nesting size Presence of secondary sexual characteristics Plastron softness	Sharks Seabirds Sea Turtles	Lemon shark (*Negaprion brevirostris*) Sandbar shark (*Carcharhinus plumbeus*) Bonnethead sharks (*Sphyrna tiburo*) Spinner shark (*C. brevipinna*) Winter skate (*Leucoraja ocellata*) Thorny skate (*Amblyraja radiata*) Porbeagle shark (*L. nasus*) Shortfin mako (*I. oxyrinchus*) Blue shark (*P. glauca*) Draughtboard shark (*Cephaloscyllium laticeps*) Whale shark (*Rhincodon typus* Royal penguin (*Eudyptes schlegeli*) Rockhopper penguin (*Eudyptes chrysocome*) Magellanic penguin (*S. magellanicus*) Blue penguin (*E. minor*) Storm petrel (*Oceanodroma furcata*) Southern giant petrel (*Macronectes giganteus*) Great black-backed gull (*Larus marinus*) American coot (*Fulica americana*) Green sea turtle (*Ch. mydas*) Loggerhead sea turtle (*C. caretta*)	Sex determination Age class determination Reproductive stage Sexual maturity determination Reproductive activity (active breeders vs. inactive sea turtle males)	Non-invasive, external indicators Inaccuracy in population structure estimates and sexual maturation Discriminant functions: Requires molecular approaches to validate discriminant functions (seabirds) Depends on: Age class Time of birth Habitat condition (wild/captive) Geographical region Visual examination: Error in interpretations when used alone Lack of external criteria for both sexes (sharks) Lack of correlation between presence of secondary sexual characteristics and size/age (sea turtles) Population differences Contradictory when compared with sex steroid levels and laparoscopic results Requires further evaluation and validation for determination of sexual maturation	Caldwell (1962); Owens *et al*. (1978); Boersma and Davies (1987); Kerry *et al*. (1992); Rasmussen and Gruber (1993); Joung and Chen (1995); Hull (1996); Musick and Limpus (1997); Zavalaga and Paredes (1997); Mawhinney and Diamond (1999); Hickerson (2000); Bertellotti *et al*. (2002); Gelsleichter *et al*. (2002); Hocken and Russell (2002); Seminoff *et al*. (2002, 2003); Setiawan *et al*. (2004); Casale *et al*. (2005); Francis and Duffy (2005); Joung *et al*. (2005); Sulikowski *et al*. (2005, 2006); Copello *et al*. (2006); Koch *et al*. (2007); Norman and Stevens (2007); Awruch *et al*. (2008a,b); Blanvillain *et al*. (2008); Lopez-Castro *et al*. (2010); Poisbleau *et al*. (2010); Labrada-Martagón *et al*. (2013)

Mark–recapture and sequential measurements of the same individuals at time intervals (Haddon, 2001; de Mitcheson, 2009) and photo-identification, based on a photographic catalogue and recognition of variations of the dorsal fin and fluke patterns (Karczmarski and Cockcroft, 1998), are the most commonly used methods for the determination of many important life history and demographic data, in both captive and live free-ranging organisms (Table 1). Photo-identification has been used as a gold standard approach to validate the conclusions based on hormone concentrations measured in cetaceans (Rolland *et al.*, 2005). Studying the relationship between the reproductive endocrinology and the reproductive breeding ecology has been favoured by tagging methods in seabirds such as penguins and albatrosses (e.g. Adélie penguin and Emperor penguin), in which the identification and monitoring of specific individual behaviours (e.g. copulatory position, arrival date at breeding colony, vocalizations, incubation and attendance patterns) and breeding conditions (e.g. incubation, moulting and reproductive success) could be used as indirect approaches to determine classification criteria, such as sex determination (e.g. Groscolas *et al.*, 1986; Hector *et al.*, 1986a,b; Williams, 1992a; Cherel *et al.*, 1994; Mauget *et al.*, 1994). The monitoring of specific individual behaviours is facilitated by the large number of organisms in a colony, the accessibility to observe marked individuals in their habitat (ice-free and open areas), their large size, flightlessness and site philopatry (e.g. Ainley and DeMaster, 1980; Ainley *et al.*, 1983; Groscolas *et al.*, 1986; Boersma *et al.*, 1990; Dann and Cullen, 1990; Darby and Seddon, 1990; Crawford *et al.*, 1999; Dugger *et al.*, 2006). However, in other marine species there are difficulties in the process of capturing and tagging individuals in particular habitats and age classes (Balazs, 1995), dealing with the low rate of recaptures (Meylan, 1995; Lopez-Castro *et al.*, 2010) or tag loss (Limpus, 1992; Balazs, 1995, 1999; Chaloupka and Musick, 1997) and the requirement for long-term monitoring mark–recapture series (e.g. more than 20 years in sea turtles; Carr, 1984; Bjorndal *et al.*, 2000; Table 1).

The examination of gonadal condition by histological methods and the creation of indices derived from them (e.g. gonadalsomatic index) have been widely used to assess the reproductive biology, reproductive condition, size at maturity and fecundity rate and for sex determination of sharks, penguins, sea turtles and marine mammals (e.g. Strasburg, 1958; Stevens, 1983; Perrin and Reilly, 1984; Hohn *et al.*, 1985; Natanson and Cailliet, 1986; Read, 1990; Kusher *et al.*, 1992; Gross *et al.*, 1995; Hazin *et al.*, 2001; Blanvillain *et al.*, 2008; Xia *et al.*, 2011). These findings lead to a variety of integrative and comparative reviews about the reproductive biology and endocrinology (hormone dynamics) of some species (e.g. Licht, 1979, 1982; Owens, 1980, 1997; Owens and Morris, 1985; Callard *et al.*, 1989; Valencia and Leyton, 1992; Hamann *et al.*, 2003). Nevertheless, some aspects of the reproductive endocrinology and ecology, such as stage duration (e.g. juveniles) and size or age at maturity, are still inconclusive for groups such as sea turtles and sharks (Rasmussen and Gruber, 1993; Musick and Limpus, 1997; Gelsleichter *et al.*, 2002; Seminoff *et al.*, 2002; Heppell *et al.*, 2003).

Laparoscopic (sea turtles and avian species) and cloacascopic examinations (penguins) are sexing techniques used in live and free-ranging immature sea turtles, chicks and adult penguins without reproductive behaviour (e.g. copulation), and have also been used to determine the reproductive condition and maturity status of sea turtles (Ainley *et al.*, 1983; Owens, 1999; Wibbels, 1999; Jessop *et al.*, 2004a; Blanvillain *et al.*, 2008). Laparoscopy is considered the most accurate method of the sexing techniques owing to the capacity to visualize the gonads directly (Wibbels *et al.*, 2000; Blanvillain *et al.*, 2008) and, together with histological examinations, has been broadly used as the gold-standard method of validation of other, less invasive sexing approaches. Cloacascopy, on contrast, requires structures well differentiated in size for successful sexing, and the characteristics of cloacae could differ between bird species, making the applicability of this tool difficult in the field (Samour *et al.*, 1983; Boersma and Davies, 1987; Costantini *et al.*, 2008). Ultrasonography is a promising external, non-invasive technique used to evaluate reproductive status based on ultrasound images of the internal organs. This tool is suggested as an alternative for the determination of the reproductive condition of sea turtles and marine mammals (dolphins); however, further studies are needed to evaluate its applicability, particularly to immature or non-reproductive sea turtles (Rostal *et al.*, 1990; Brook *et al.*, 2000; Brook, 2001; Blanvillain *et al.*, 2008). This technique has not been useful to identify the sex of immature loggerhead sea turtles, owing to the small size of the undeveloped gonads (Valente *et al.*, 2007). As a result of the similarity between the general appearance of the reproductive structures described by ultrasound in active male dolphins and sea turtles (for which comparative data exist) and some correlations found between the measurements taken of the reproductive organs of adult male loggerhead sea turtles with laparoscopy and ultrasonography, such as the epididymal duct diameter (Pearson's *r* = 0.91, *P* < 0.001), it is possible that ultrasonography may ultimately replace the laparoscopic procedure (Blanvillain *et al.*, 2008).

Biometric data (e.g. bill, foot, wing and vent measurements) and comparison of phenotypic characteristics (e.g. clasper morphology, tail size, mean nesting size and plastron softness) are non-invasive, discriminant methods to assess demographic parameters, such as reproductive stage and age/sex determination (Table 1). In sharks, external secondary sexual characteristics of clasper (length, rotation and calcified condition), visual signs of mating and courtship (scars, sperm in clasper and haematoma around the cloacae) and length, when the size at maturity is known for the species, have been used, together with other histological and endocrine approaches, as a sexual discriminant criterion and to assess the maturity of males (e.g. Rasmussen and Gruber, 1993; Joung and Chen, 1995; Gelsleichter *et al.*, 2002; Francis and Duffy, 2005; Joung *et al.*, 2005; Sulikowski *et al*, 2005, 2006; Norman and Stevens, 2007; Awruch *et al.*, 2008a,b). In the threatened whale shark (*Rhincodon typus*), the sex of 85% of the individuals observed off the coast of Australia (*n* = 325) was identified, while diving with the animal, by clasper presence, and sexual maturity was assessed in the total of males found (100%), confirming that clasper condition criterion is a visual, non-invasive approach to assess demographic parameters for this species (Norman and Stevens, 2007). Nevertheless, an external visual criterion to assess female sexual maturity does not exist in sharks (Francis and Duffy, 2005; Norman and Stevens, 2007; Awruch *et al.*, 2008a).

Softness of the plaston has been suggested as a visual criterion to identify active male breeders from immature sea turtles; nevertheless, the T concentration measured in plasma has not shown a significant correlation with the percentage relative plastron softened area, and additional studies are needed (Blanvillain *et al.*, 2008). The mean nesting size and the tail size are parameters widely used to define the adult stage, for both female and male sea turtles (Musick and Limpus, 1997; Seminoff *et al.*, 2002, 2003; Casale *et al.*, 2005; Koch *et al.*, 2007; Lopez-Castro *et al.*, 2010); there is no external visual criterion for sexing juvenile stages. However, size and the absence of secondary sexual characteristics (e.g. nails and tail development in male sea turtles) are not good indicators of the sex and reproductive stage when used alone as classification criteria in feeding grounds, where large immature and male adult sea turtles are found mixed together (Caldwell, 1962; Owens *et al.*, 1978). Growth models suggest that sea turtles reach sexual maturity at a size larger than the estimated mean nesting size (Seminoff *et al.*, 2002). Even when sea turtle species exhibit a common delayed maturity (Heppell *et al.*, 2003), the size of these organisms is not a reliable indicator of maturity, because populations of the same species (e.g. green turtle; Zug *et al.*, 1986; Green, 1993; Balazs, 1995; Seminoff *et al.*, 2002) do not start to breed at the same age or minimal size (Zug *et al.*, 1986; Miller, 1997). Laparoscopic and steroid-based studies suggest that the mean nesting size could mislead estimates of population structure, with potential underestimation of the number of males (Limpus *et al.*, 1994; Miller, 1997; Labrada-Martagón *et al.*, 2013).

Despite the great sex classification power of the discriminant analysis obtained from biometric data on seabirds (84–100%; Boersma and Davies, 1987; Kerry *et al.*, 1992; Hull, 1996; Mawhinney and Diamond, 1999; Hocken and Russell, 2002; Copello *et al.*, 2006), a variety of canonical discriminant functions (biometric classification parameters) has been suggested, between reproductive stages (e.g. flipper length in immature Magellanic penguins (*Spheniscus magellanicus*) vs. bill length in adults; Bertellotti *et al.*, 2002) and between seabird species, making this approach applicable for all age classes (immature), time of birth (chicks) and conditions (wild vs. captive) difficult without previous validation (Table 1). However, this has to be interpreted with caution when used with data from other geographical populations (Hull, 1996; Zavalaga and Paredes, 1997; Bertellotti *et al.*, 2002; Setiawan *et al.*, 2004; Copello *et al.*, 2006; Poisbleau *et al.*, 2010). Currently, molecular techniques using non-invasive (eggs and feathers) or non-lethal samples (blood) give the most reliable and accurate method for sexing avian species (Bertellotti *et al.*, 2002; Bush *et al.*, 2005; Cerit and Avanus, 2007; for additional reviews see Seutin *et al.*, 1991; Jensen *et al.*, 2003; Bush *et al.*, 2005; Dubiec and Zagalaska-Neubauer, 2006).

## Conclusions

The limitations in pooled analyses include heterogeneity between studies and the different methods of recruiting the controls used (Raimondi *et al.*, 2006). Thus, it is difficult to compare the accuracy and classification power of different approaches used to obtain life history data in wildlife (e.g. biometric data and hormone concentrations), owing to the heterogeneity of the information reported between studies (e.g. means, ranges, percentage of accuracy and correlation coefficients) and the classification criteria used (e.g. sex, age class and reproductive stages), in addition to the specific differences in this review (e.g. sharks, seabirds and sea turtles). Another limitation during the sensitivity analysis, performed on those published reports focusing on validation of the use of hormone concentrations, was the variety of samples (serum, plasma, amniotic fluid and faeces) and validation approaches or controls (e.g. histology, laparoscopy, cloacascopy and photo-identification) used between studies.

Histology (sharks, sea turtles and marine mammals), laparoscopy (sea turtles), laparotomy and cloacascopy (seabirds) are the most accurate methods for determining the sex and reproductive condition of the animals, and they have been considered as the reference method (gold standard) to evaluate less-invasive approaches, such as the hormone concentrations. The main disadvantages of the classical direct approaches are logistical complications (e.g. cost, specialized equipment required and dependence on field conditions) and their invasive or lethal characteristics when applied to threatened or endangered species. Photo-identification (marine mammals) and biometric (seabirds), phenotypic (sharks) and behavioural data (seabirds) are non-invasive, external validation methods of sex and reproductive stages, useful only when the information is available (e.g. known size at maturity and clasper condition); they require monitoring studies of the population through the breeding season (e.g. penguins and albatrosses) and expensive, long time series (e.g. 25 years of life history data for cetaceans). Using phenotypic characteristics of sea turtles is difficult for immature individuals and when adults are together with sub-adults. The large discriminant accuracy of biometric data when used for sexing seabirds is limited by considerable intra-specific (e.g. age class, reproductive condition and geographical population) and inter-specific variation.

The measurement of steroid hormone concentrations has shown great accuracy as a discriminant (71–100% accuracy) of sex and reproductive status (when data could be compared), with consistent accuracy between samples (e.g. amniotic fluid and plasma) and studies (Gross *et al.*, 1995; Xia *et al.*, 2011), thus demonstrating the reproducibility and diagnostic validity of this method. The advantages of hormone determination as a non-lethal and less-invasive tool for the generation of valuable demographic information needed for stock assessment and biological conservation include low cost, the facility for sample preservation, and little time and effort employed in both field and laboratory conditions. Validation of such data is needed in specific populations in order to guarantee the accurate interpretation of subsequent hormone determinations (Wibbels, 1999). There are many descriptive studies on the reproductive endocrinology (the relationship between hormone concentrations and reproductive behaviour) of marine species. A large number of associations have already been demonstrated between sex steroid concentrations and biochemical parameters, gonadal state, reproductive behaviour and secondary external features (e.g. clasper in sharks). However, there is a lack of information about direct quantitative correlations between the gonadal changes observed using lethal methods and the hormone concentrations measured in the individuals (e.g. Wibbels *et al.*, 1990; Sulikowski *et al.*, 2005, 2006; Blanvillain *et al.*, 2008), even when the information was available, and there are even fewer studies that have proposed some quantitative approach to discriminate demographic parameters based on hormone concentrations (Awruch *et al.*, 2008a).

In addition to the use of hormone concentrations as a sexing method, the most recent studies on sharks (Sulikowski *et al.*, 2005, 2006, 2012; Awruch *et al.*, 2008a) have suggested that measurements of circulating sex steroids can be used as an indirect approximation to assess sexual maturity and reproductive condition in those species without evident secondary sexual characteristics and in the absence of morphological data (e.g. gonadal condition). Clearly, the relationship between plasma hormone changes during puberty and the sexual maturation process could be studied in other marine vertebrate groups, by comparing sex steroid concentrations by age/size classes at pre-mature stages and between groups with or without evident secondary sexual characteristics. This would allow validation of the size classes and pre-mature stages and/or conditions at which hormone determination provides accurate demographic information. The link between deferred sexual maturity and circulating steroid concentrations needs further clarification. Understanding the dynamics of the hormone concentrations during puberty and sexual maturity allows for identification of sex, stage duration, size range at maturation and reproductive condition, expanding the information derived from external characteristics and morphological measurements when used alone. For example, in skates the simultaneous evaluation of multiple techniques has demonstrated that hormone determinations provide more precise estimates of sexual maturity stage (expressing a lag period between gonadal development and functional maturity) than the morphological measurements alone (Sulikowski *et al.*, 2006). In the East Pacific population of green sea turtles, hormone determination for immature turtles suggests a bias in the estimation of the population structure when considering the mean nesting size as a unique classification criterion (overestimation of adult females due to the absent secondary sexual characteristics expected in males; Labrada-Martagón *et al.*, 2013).

We lack information about demographic data in many marine species and populations that are the focus of conservation interest. The current information available about reproductive endocrinology, stage duration (growth) and age/size at sexual maturity is centred on a few populations (e.g. green sea turtles from Hawaii, Florida and Australia), habitats (nesting grounds) and reproductive stages (adults) in some species. Consequently, key demographic parameters used to establish policies and conservation strategies remain unknown. The information derived from sex steroid concentrations in different stages and size classes (Rasmussen and Gruber, 1993; Awruch *et al.*, 2008a; Sulikowski *et al.*, 2012), together with other phenotypic (e.g. total length and clasper length; Awruch *et al.*, 2008a) and biochemical parameters that have explained the steroid concentrations measured in immature sea turtles (e.g. glucose, cholesterol and thyroxine levels; Labrada-Martagón *et al.*, 2013) and energy allocation patterns in adult females (triglycerides; Hamann *et al.*, 2002a,b) form the basis for future determinations on demographic data (e.g. sex and reproductive stage), population structure and seasonal variations in the development and reproductive condition of the populations as well.
